# Protocol for a systematic review of the application of the kidney failure risk equation and Oxford classification in estimating prognosis in IgA nephropathy

**DOI:** 10.1186/s13643-024-02543-y

**Published:** 2024-05-04

**Authors:** Michael Toal, Ruth Fergie, Michael Quinn, Christopher Hill, Ciaran O’Neill, Alexander P. Maxwell

**Affiliations:** 1 Centre for Public Health, Queen’s University of Belfast, Belfast, BT12 6AB Northern Ireland; 2 Regional Nephrology Unit, Belfast City Hospital, Lisburn Road, BT9 7BA Belfast, Northern Ireland

**Keywords:** IgA nephropathy, Glomerulonephritis, Renal biopsy, Chronic kidney disease, Disease progression, Kidney failure, End-stage renal disease, Prediction tool, Kidney Failure Risk Equation, Oxford classification

## Abstract

**Background:**

IgA nephropathy (IgAN) is a common cause of chronic kidney disease (CKD) and end-stage renal disease (ESRD). Outcomes are highly variable and predicting risk of disease progression at an individual level is challenging. Accurate risk stratification is important to identify individuals most likely to benefit from treatment. The Kidney Failure Risk Equation (KFRE) has been extensively validated in CKD populations and predicts the risk of ESRD at 2 and 5 years using non-invasive tests; however, its predictive performance in IgAN is unknown. The Oxford classification (OC) describes pathological features demonstrated on renal biopsy that are associated with adverse clinical outcomes that may also inform prognosis. The objective of this systematic review is to compare the KFRE with the OC in determining prognosis in IgAN.

**Methods:**

A systematic review will be conducted and reported in line with PRISMA guidelines (PRISMA-P checklist attached as Additional file 1). Inclusion criteria will be cohort studies that apply the KFRE or OC to determine the risk of CKD progression or ESRD in individuals with IgAN. Multiple databases will be searched in duplicate to identify relevant studies, which will be screened first by title, then by abstract and then by full-text analysis. Results will be collated for comparison. Risk of bias and confidence assessments will be conducted independently by two reviewers, with a third reviewer available if required.

**Discussion:**

Identifying individuals at the highest risk of progression to ESRD is challenging in IgAN, due to the heterogeneity of clinical outcomes. Risk prediction tools have been developed to guide clinicians; however, it is imperative that these aids are accurate and reproducible. The OC is based on observations made by specialist renal pathologists and may be open to observer bias, therefore the utility of prediction models incorporating this classification may be diminished, particularly as in the future novel biomarkers may be incorporated into clinical practice.

**Systematic review registration:**

PROSPERO CRD42022364569

**Supplementary Information:**

The online version contains supplementary material available at 10.1186/s13643-024-02543-y.

## Background

IgA nephropathy (IgAN) was first described by Berger and Hinglais in 1968 and is the most common form of primary glomerulonephritis worldwide [1]. The disease course of IgAN is heterogeneous with up to 30–50% of affected individuals progressing to end-stage renal disease (ESRD) within 5 to 10 years, whilst others have stable disease without significant progression over decades [2–4]. Identifying individuals at the highest risk for progression to ESRD is challenging and the 2021 Kidney Disease Improving Global Outcomes (KDIGO) guidelines highlighted risk stratification as a high priority for research in IgAN [5]. The understanding of the pathophysiology of IgAN is constantly evolving and there are multiple therapeutic trials ongoing to find novel agents to stabilise patients at the highest risk of progression to ESRD [6, 7]. Previous therapeutic trials in IgAN have been limited by the recruitment of individuals at lower risk of progression to ESRD. Timely risk stratification to identify persons most likely to progress to ESRD will allow for more targeted clinical trials in the future [8, 9].

A renal biopsy is a crucial investigation in nephrology that facilitates the diagnosis of kidney diseases, estimation of prognosis and guides treatment [10]. It is however an invasive procedure, with a significant risk of bleeding, requiring intervention in around 1 in 200 procedures in some case series [11, 12]. The Oxford classification (OC) is a histopathological scoring system developed to facilitate estimations of prognosis by classifying the degree of pathological abnormalities demonstrated on renal biopsy examination and is specific to IgAN [13]. The IgAN Risk Prediction (IRP) tool was developed specifically for IgAN and incorporates the OC into the risk evaluation (Table 1) [14]. The Kidney Failure Risk Equation (KFRE) (Table 1) was developed from a Canadian cohort and validated in a multi-national cohort of over 700,000 patients [15]. This equation, using either four or eight variables, has demonstrated excellent discrimination and can accurately predict the risk of kidney failure at a 2- or 5-year horizon for all forms of chronic kidney disease (CKD).

**Table 1:** Parameters required for each prognostic algorithm

Variable	KFRE	OC	IRP
Sex	✔		
Age	✔		✔
Race			✔
Location	✔		
eGFR	✔		✔
Proteinuria^a^	✔		✔
MAP			✔
M^b^		✔	✔
E^b^		✔	✔
S^b^		✔	✔
T^b^		✔	✔
C^b^		✔	
RASB use at time of biopsy			✔
Immunosuppression use at/prior to biopsy			✔
Outcome	Risk of kidney failure at 2 and 5 years		Risk of composite outcome of 50% decline in eGFR and kidney failure at 5 years

*KFRE* kidney failure risk equation, *OC* Oxford classification, *IRP* IgAN Risk Prediction tool, *eGFR* estimated glomerular filtration rate (ml/min/1.73 m^2^), *MAP* mean arterial pressure (mmHg), *M* mesangial hypercellularity score, *E* endocapillary hypercellularity score, *S* segmental sclerosis score, *T* tubular atrophy/interstitial fibrosis score, *C* crescents score, *RASB* renin-angiotensin system blockers

^a^Proteinuria expressed as urinary albumin:creatinine ratio in KFRE and grams/day in IRP

^b^Obtained by renal biopsy

Whilst the OC, IRP and KFRE have been externally validated, there is limited evidence to determine which method provides optimal prognostic assessment, therefore it is unknown if a renal biopsy improves the accuracy of this estimation. Affected individuals will often have multiple blood and urine tests over a follow-up period; however, an invasive renal biopsy is seldom repeated. The OC will therefore provide a snapshot of disease activity but does not capture the nuances and dynamic changes in disease trajectory, which can be routinely tracked with blood and urine tests. If the OC did not provide superior predictive accuracy compared to clinical features alone, the international community may reconsider the utility of the OC in renal biopsy reports of IgAN.

## Objectives

The research question for this review is do models incorporating the Oxford Classification (MEST-C score) provide a more accurate prediction of disease progression and kidney failure than the Kidney Failure Risk Equation in patients with IgAN?

## Methods

### Review registration

This research was designed in accordance with Preferred Reporting Items for Systematic Reviews and Meta-Analysis Protocols (PRISMA) and registered on the International Prospective Register of Systematic Reviews on 4th October 2022 (PROSPERO no. CRD42022364569). This protocol was started on 5th September 2022 and the completed review was submitted for publication on 27th October 2023 and is currently awaiting peer review.

### Eligibility criteria

Studies that will be considered for inclusion in the review will be those which investigate a population or sub-group of patients diagnosed with IgAN on native renal biopsy and compare the prognostic accuracy of the four- or eight-variable Kidney Failure Risk Equation (KFRE) with an alternative prediction tool which incorporates the OC of histopathological assessment. The inclusion criteria for study outcomes will include progression of disease as defined by a reduction in estimated glomerular filtration rate (eGFR), rise in serum creatinine or kidney failure events as defined by an eGFR below 15 ml/min/1.73 m^2^ or use of renal replacement therapies (kidney transplantation or dialysis). The KFRE was first published in 2011; therefore, studies from 2011 onwards will be considered for inclusion. Research subjects must be humans only. Eligibility criteria will not be limited to the English language and translation services will be used where appropriate. Publications will not be limited to journal articles and appropriate grey literature will be considered eligible.

### Information sources

The following databases will be searched: Medline, EMBASE, Scopus, Web of Science and Google (including Google Scholar).

### Search strategy

The detailed search strategy for each database is reported in Additional file 2: Appendix 1. The following keywords will be used across all databases:

1. “IgA Nephropathy” OR “Immunoglobulin A Nephropathy” OR “Glomerulonephritis, IGA” OR “Berger’s disease”.
2. “Kidney Failure Risk Equation” OR “KFRE”.
3. “Oxford” OR “MEST” OR “MEST-C”.
4. “Disease progression” OR “Prognostic Assessment” OR “Risk Assessment”.
5. 1 AND 2 AND 3 AND 4

### Study records

Study records will be imported into reference management software (Rayyan™) which will remove duplicates and maintain a library of eligible studies. Screening of studies will be performed independently by the corresponding author (MT) and a second reviewer (RF). Any disagreements regarding the suitability of studies will be discussed amongst the two reviewers and a third reviewer (APM), if necessary. Studies will initially be screened by title, then by abstract and then by reviewing the full-text article.

Data will be extracted using a standardised form for collating study data. The study authors will be contacted by MT if crucial data is missing in the eligible studies that prohibit analysis.

### Data items

The review will be limited to patients with a diagnosis of IgAN, proven by a native renal biopsy. Patients with all other diagnoses based on renal biopsy will not be included, nor will patients with other forms of renal diseases, not diagnosed by renal biopsy. Any international cohort or sub-group will be considered eligible. Measurements of proteinuria will be converted into standardised units of albumin: creatinine ratio where required [16].

### Outcomes and prioritisation

Studies that include measurements of discrimination and calibration such as the area under the curve (AUC) of the receiver-operating curve or Harrell’s C-statistic will be included. Studies that assess calibration using predicted outcomes and observed outcomes will also be eligible for inclusion. Excluded studies will be those which do not report either of the aforementioned outcomes and are limited to other parameters such as relative risk and hazard ratios without a direct measure of classification. Follow-up time of the cohort equal to or beyond 2 years from the renal biopsy will be considered to be appropriate for inclusion in the analysis.

### Risk of bias in individual studies

The risk of bias will be assessed in any study meeting the inclusion criteria using PROBAST (Prediction model Risk Of Bias ASsessment Tool) [17]. This tool was designed specifically for studies of prognostic assessment, as many different prediction methods can be available for single disease entities. It comprises twenty signalling questions across four domains: participants, predictors, outcomes and analysis. For each domain, the reviewer is required to determine a risk of bias and a level of concern regarding the study’s applicability to the review question. Two independent reviewers (MT and RF) will use PROBAST to assess the risk of bias, with APM acting as a third reviewer if required.

### Data synthesis

Data from eligible studies will be collated for meta-analysis if possible. The number of participants in each cohort study will be described and their geographical location will be included. The baseline characteristics of all participants will be described in terms of age, gender and measurements of kidney function such as eGFR, serum creatinine and proteinuria. The long-term outcomes of each cohort will also be described—specifically detailing the incidence of ESRD. The prognostic accuracy of tools under investigation will be described using Harrell’s c-index, a derivation from the AUC. These results will be collated into a graphical form for comparison. Heterogeneity in studies will be assessed using the I^2^ method. A subgroup analysis will investigate prognostic accuracy for studies that included other outcome measures such as a decline in eGFR over time of 30%, 40% or 50%.

### Meta-bias

The risk of publication bias will be examined by evaluation of individual patient data provided by studies. If this is not adequately described in a paper, the authors will be contacted in order to ensure there is transparency of results without selective reporting. A funnel plot will be employed to investigate the risk of publication bias by demonstration of asymmetry, with a *p* < 0.10 considered to be significant [18]. Reviewer selection bias will be addressed by two independent reviewers performing an identical search strategy, with a third reviewer available to resolve any inconclusive results.

### Confidence in cumulative evidence

The certainty in the evidence presented will be analysed using the GRADE approach. This assessment analyses the quality of five domains: risk of bias, inconsistency, imprecision, indirectness and publication bias [19]. This system will be applied to each described outcome, detailing the authors’ confidence in each conclusion. Recommendations will be given for future studies to address the current gaps in the literature.

## Discussion

The burden of CKD is increasing globally and is projected to become the 5th most common cause of years of life lost by 2040 [20]. IgAN is a common cause of CKD which has been observed to progress to ESRD in up to 50% of cases within 5 years of follow-up in a UK cohort study [4]. The increased risk of mortality has also been observed specifically in an IgAN cohort study, with progression to ESRD associated with a threefold increase in mortality within the follow-up period (median 13.6 years) [21]. There are numerous available risk prediction tools for kidney disease; however, the KFRE and OC were selected for this review as they have been extensively validated and incorporated into UK and international guidelines [5, 22].

The OC has been adopted internationally since its conception in 2009; however, the reported prognostic value of each component of the MEST-C score varies in the literature and poor reproducibility between reporting pathologists has been identified as a significant limitation [23–25]. The KFRE is based on age, sex, location and automated laboratory values, therefore it may have increased reproducibility, but its application in IgAN has not been investigated in detail.

As the understanding of the pathophysiology in IgAN continues to improve, therapeutic targets are likely to become more readily available to manage individuals at higher risk of progression [26]. Several biomarkers have been identified in IgAN for diagnosis and prognostic assessment; however, these have not yet come into clinical practice [27, 28]. In membranous nephropathy, the highly specific anti-phospholipase A2 receptor antibody test has the potential to mitigate the requirement for renal biopsy and expose individuals to the hazards associated [29]. If such a biomarker for IgAN becomes integrated into clinical practice, diagnostic and prognostic approaches may need to adapt towards more non-invasive methods.

The outcomes associated with IgAN are heterogeneous, therefore identifying high-risk individuals who require close monitoring and treatment is challenging. This review is intended to assist clinicians in selecting the most accurate tool to provide effective and tailored treatment to individuals affected by IgAN.

### Supplementary Information

**Additional file 1.** PRISMA-P 2015 Checklist.

**Additional file 2. Appendix 1.** Search strategy.

## Data Availability

Not applicable.

## Abbreviations

AUC: Area under the curve
CKD: Chronic kidney disease
eGFR: Estimated glomerular filtration rate
ESRD: End-stage renal disease
GRADE: Grades of Recommendation, Assessment, Development and Evaluation Working Group
KDIGO: Kidney Disease Improving Global Outcomes
KFRE: Kidney failure risk equation
IgAN: Immunoglobulin A nephropathy
IRP: IgAN Risk Prediction tool
MEST-C: Mesangial hypercellularity, Endocapillary hypercellularity, Segmental sclerosis, Tubular atrophy/interstitial fibrosis, Crescents
PROBAST: Prediction model Risk of Bias Assessment tool
OC: Oxford classification
RRT: Renal replacement therapies
