# Supplementary material for: Protocol for a systematic review of the application of the kidney failure risk equation and Oxford classification in estimating prognosis in IgA nephropathy
Source: Syst Rev. 2024 May 4;13:122. doi: 10.1186/s13643-024-02543-y (PMC11070080; doi:10.1186/s13643-024-02543-y)
Supplement: Supplementary file 2 — Additional file 2. Appendix 1. Search strategy. [file 13643_2024_2543_MOESM2_ESM.docx]

Appendix 1: Search Strategy

MEDLINE

| 1 | “IgA Nephropathy” OR “Immunoglobulin A Nephropathy” OR “Glomerulonephritis, IGA” OR “Berger$” |
| --- | --- |
| 2 | “Kidney Failure Risk Equation” OR “KFRE” |
| 3 | “Oxford” OR “MEST” OR “MEST-C” |
| 4 | “Disease progression” OR “Prognostic Assessment” OR “Risk Assessment” |
| 5 | 1 AND 2 AND 3 AND 4 |
| 6 | Limit 5 to year “2011-present” |

EMBASE

| 1 | “IgA Nephropathy” OR “Immunoglobulin A Nephropathy” OR “Glomerulonephritis, IGA” OR “Berger$” |
| --- | --- |
| 2 | “Kidney Failure Risk Equation” OR “KFRE” |
| 3 | “Oxford” OR “MEST” OR “MEST-C” |
| 4 | “Disease progression” OR “Prognostic Assessment” OR “Risk Assessment” |
| 5 | 1 AND 2 AND 3 AND 4 |
| 6 | Limit 5 to year “2011-present” |

SCOPUS

| 1 | “All fields” | “IgA Nephropathy” OR “Immunoglobulin A Nephropathy” OR “Glomerulonephritis, IGA” OR “Berger$” |
| --- | --- | --- |
| 2 | “All fields” | “Kidney Failure Risk Equation” OR “KFRE” |
| 3 | “All fields” | “Oxford” OR “MEST” OR “MEST-C” |
| 4 | “All fields” | “Disease progression” OR “Prognostic Assessment” OR “Risk Assessment” |
| 5 |  | 1 AND 2 AND 3 AND 4 |
| 6 |  | Limit 5 to year “2011-present” |

WEB OF SCIENCE

| 1 | “All fields” | “IgA Nephropathy” OR “Immunoglobulin A Nephropathy” OR “Glomerulonephritis, IGA” OR “Berger$” |
| --- | --- | --- |
| 2 | “All fields” | “Kidney Failure Risk Equation” OR “KFRE” |
| 3 | “All fields” | “Oxford” OR “MEST” OR “MEST-C” |
| 4 | “All fields” | “Disease progression” OR “Prognostic Assessment” OR “Risk Assessment” |
| 5 |  | 1 AND 2 AND 3 AND 4 |
| 6 |  | Limit 5 to year “2011-present” |

GOOGLE SCHOLAR

| 1 | “IgA Nephropathy” |
| --- | --- |
| 2 | “Kidney Failure Risk Equation” |
| 3 | “Oxford” |
| 4 | 1 AND 2 AND 3 |
| 5 | Limit 5 to year “2011-present” |
